# Three-dimensional alignment of microvasculature and cardiomyocytes in the developing ventricle

**DOI:** 10.1038/s41598-020-71816-y

**Published:** 2020-09-11

**Authors:** Maryse Lapierre-Landry, Hana Kolesová, Yehe Liu, Michiko Watanabe, Michael W. Jenkins

**Affiliations:** 1 Department of Biomedical Engineering, School of Medicine, Case Western Reserve University, Wood Building WG28, 2109 Adelbert Road, Cleveland, OH 44106 USA; 2 Department of Pediatrics, School of Medicine, Case Western Reserve University, Cleveland, USA; 3 Institute of Anatomy, First Faculty of Medicine, Charles University, Prague, Czech Republic

**Keywords:** Cardiovascular biology, Biomedical engineering

## Abstract

While major coronary artery development and pathologies affecting them have been extensively studied, understanding the development and organization of the coronary microvasculature beyond the earliest developmental stages requires new tools. Without techniques to image the coronary microvasculature over the whole heart, it is likely we are underestimating the microvasculature’s impact on normal development and diseases. We present a new imaging and analysis toolset to visualize the coronary microvasculature in intact embryonic hearts and quantify vessel organization. The fluorescent dyes DiI and DAPI were used to stain the coronary vasculature and cardiomyocyte nuclei in quail embryo hearts during rapid growth and morphogenesis of the left ventricular wall. Vessel and cardiomyocytes orientation were automatically extracted and quantified, and vessel density was calculated. The coronary microvasculature was found to follow the known helical organization of cardiomyocytes in the ventricular wall. Vessel density in the left ventricle did not change during and after compaction. This quantitative and automated approach will enable future cohort studies to understand the microvasculature’s role in diseases such as hypertrophic cardiomyopathy where misalignment of cardiomyocytes has been observed in utero.

## Introduction

The coronary microvasculature is an extensive vessel network that serves the constantly contracting cardiomyocytes by providing nutrients and oxygen while removing waste products. The large coronary vessels play a significant role in many serious congenital and adult diseases^1–3^. The coronary microvasculature is also now being implicated in many diseases including congenital heart disease (CHD). For example, swollen coronary sinusoids have been detected in children with pulmonary atresia^4,5^ and patients with d-transposition of the great arteries and tetralogy of Fallot have reduced myocardial perfusion via the microvasculature^6^. Patients with CHDs often have reduced exercise tolerance, which could point toward microvascular defects^7^. However, expansion in our understanding of microvascular heart development and its role in heart disease requires new tools and a quantitative approach.

The coronary microvasculature forms via a complex process^8–13^. Coronaries first appear as a plexus within the epicardium that forms by vasculogenesis and matures into a branching network by a combination of vasculogenesis, angiogenesis and arteriogenesis once connections to the aorta are established. Smaller coronary branches are not restricted to the epicardium and appear within the myocardium. The smallest vessels are attached to the extracellular matrix adjacent to cardiomyocytes. How this extensive microvasculature develops after the coronary plexus stage and establishes its mature arrangement during myocardial wall morphogenesis has not been investigated.

Coronary microvasculature’s role in cardiac diseases is poorly understood due to a lack tools. There are no clinical tools that directly assess coronary microvasculature morphology and function^14,15^. Thus, we are likely underestimating the coronary microvasculature’s impact in various diseases. In research studies, there have been several strategies to visualize the microvasculature including genetically engineered markers^16–18^, antibodies to endothelial-specific markers^19–21^, and contrast agents perfused into the vasculature^17,22–26^. However, most studies yielded qualitative results and either did not visualize the entire vasculature of the developing heart or provided limited opportunity for quantification. Therefore, there is a need for comprehensive imaging and quantification of the microvasculature in the intact developing heart to facilitate high throughput cohort studies of development and disease.

Coronary microvascular orientation and its arrangement with respect to cardiomyocytes throughout the ventricular wall has not been studied. Arpino et al.^27^ demonstrated that microvascular alignment with skeletal muscle is critical for appropriate perfusion which suggests that coronary microvascular alignment with cardiomyocytes is similarly important. Qualitative data supports this co-alignment in the heart, but thorough quantitative analysis is lacking^28^. Microscopy^29–32^ and diffusion tensor cardiac magnetic resonance^33–35^ have established that cardiac muscle fibers are arranged in a helical fashion around the left ventricle, with the helical angle progressively changing from the epicardium to the endocardium. Perturbations in this arrangement are associated with disease. Cardiomyocyte disarray is one of the hallmarks of hypertrophic cardiomyopathy, a disease associated with sudden cardiac death^32,36,37^. Is cardiomyocyte disarray associated with disorganized vasculature? In our group’s previous study, coronary microvascular disarray was observed in a quail model of prenatal alcohol exposure^23^, but this was not quantified. Disorganization of the coronary microvasculature and cardiomyocytes early in development could lead to improper oxygenation and abnormal cardiac function, which are associated with further dysmorphology and dysfunction^38^.

We introduce an imaging toolset to visualize and analyze coronary microvasculature through the thickness of the quail embryo’s ventricular free wall. This toolset includes quantitative methods to measure both coronary vessel and cardiomyocyte orientation within the same hearts. We found that coronary microvasculature and cardiomyocytes were highly aligned through the depth of the left ventricle, and that the organization of the microvessels in the embryo was consistent with the known helical structure of the cardiac muscle fibers in humans^33^. This microvascular organization was consistent across multiple embryonic hearts at two developmental stages during and after the ventricular wall morphogenesis (e.g. compaction). Our findings provide a quantitative baseline for comparison of alignment in disease models and demonstrate the potential of our imaging and analysis toolset for developmental research.

## Results

### A toolset to understand coronary microvasculature and cardiomyocytes organization

**Figure 1:**
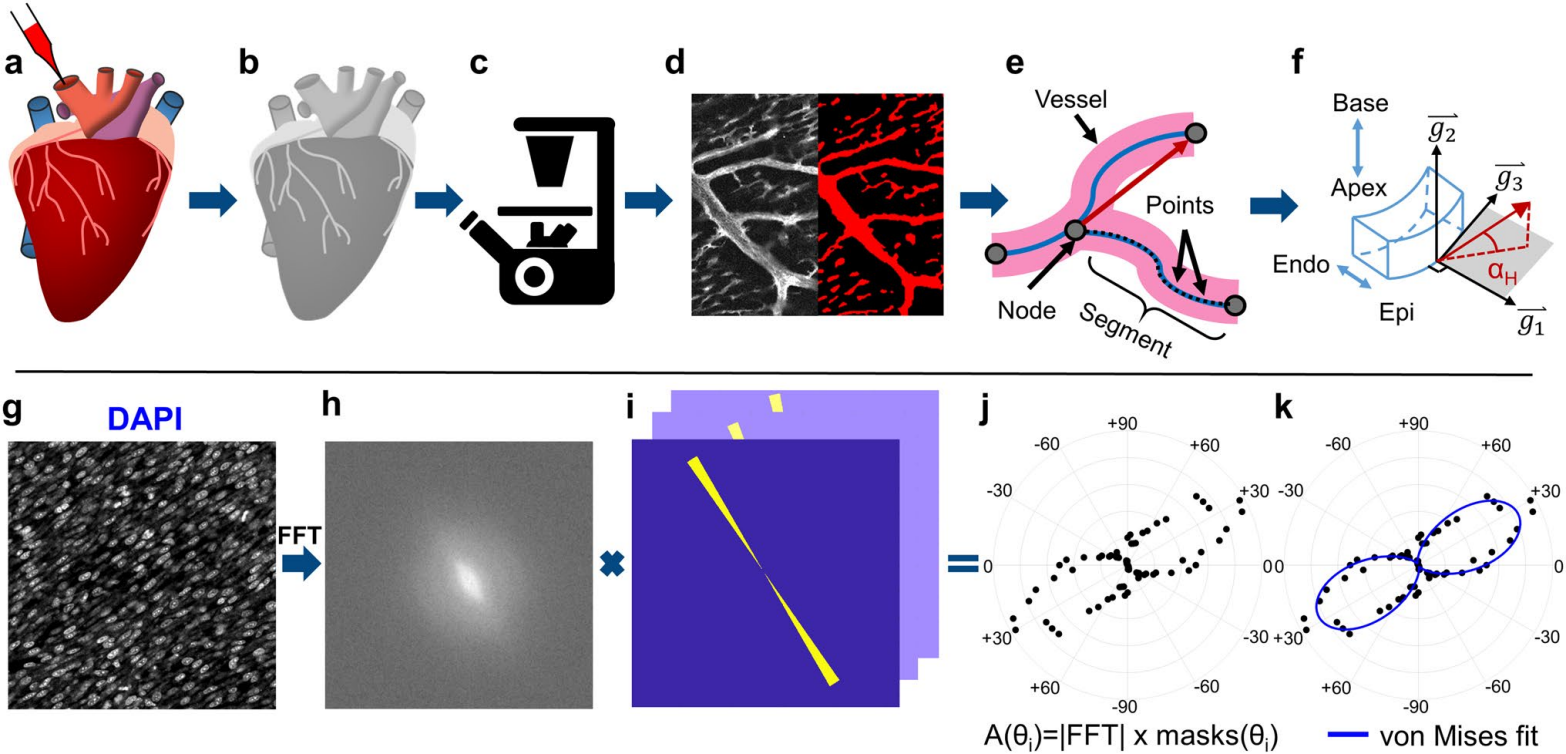
Methods summary. **(a)** Quail hearts were perfused with DiI, **(b)** optically cleared, **(c)** and imaged using confocal microscopy to detect both coronary microvasculature and cardiac nuclei. **(d)** Vessels were segmented, **(e)** and automatically skeletonized resulting in vessel-nodes, -segments and -points exported for analysis. A vessel-vector (red arrow) was defined for each vessel by connecting the start and end nodes. **(f)** The helical angle (*α*_*H*_) was calculated for each vessel-vector based on its position with respect to the heart’s surface. **(g)** Cardiac nuclei were imaged with DAPI. **(h)** A Fourier transform (FFT) was applied to each image and the resulting spectrum was multiplied by **(i)** a series of masks with different axial angles. **(j)** The resulting angular amplitude A(θ_i_) was plotted as a function of angle, **(k)** and fit to a von Mises distribution.

A solubilized lipophilic dye (DiI) was perfused into the coronary vessels via the aorta to stain the vasculature through the entire thickness of the embryonic heart (Fig. 1a). This procedure was performed at two stages with different maturation in a quail embryo model after coronary circulation had been established (embryonic day 9 and 13)^39,40^. The hearts were then optically cleared using a new optical clearing technique, termed lipid-preserving index matching for prolonged imaging depth (LIMPID) developed by our group^41^ and imaged using confocal microscopy (Fig. 1b,c).

Following imaging, the coronary vessels were automatically segmented and skeletonized (Fig. 1d,e), and each vessel helical angle (*α*_*H*_) was calculated (Fig. 1f). The helical angle was chosen to describe vessel orientation since it is often used when describing the alignment of cardiac muscle fibers^33^. To determine each vessel’s helical angle, the reference unit vectors *g*_*1*_, *g*_*2*_, *g*_*3*_ were calculated for each vessel-segment based on the direction normal to the heart surface (*g*_*1*_) the direction running from heart apex to base (*g*_*2*_), and a circumferential direction (*g*_*3*_) orthogonal to *g*_*1*_ and *g*_*2*_ (Fig. 1f). The helical angle (*α*_*H*_) was calculated for all vector segments as the elevation angle from the horizontal (*g*_*1*_*–g*_*3*_) plane (Fig. 1f) as was previously done in other studies of the heart structure^32^.

The cardiomyocyte nuclei were stained with DAPI to compare microvascular organization with the surrounding cardiac cells during rapid ventricular wall proliferation and thickening. Images of cardiac nuclei (Fig. 1g) obtained in the same hearts as the vascular images were processed using a two-dimensional (2D) Fourier transform (Fig. 1h–j) and fit to a von Mises distribution to determine nuclei orientation (Fig. 1k).

The von Mises distribution characterizes angular distribution of random processes and was used to describe our vascular and nuclei data. A bimodal von Mises probability distribution function (pdf) as a function of angle θ (in radians) is as follows^42^:

1 $$pdf(\theta ) = \frac{1}{{2\pi I_{0} (\kappa )}}e^{\kappa \cos (\theta - \mu )} + \frac{1}{{2\pi I_{0} (\kappa )}}e^{\kappa \cos (\theta + \pi - \mu )}$$

where *κ* measures the concentration of the distribution around its mean (the inverse of the dispersion), *μ* is the circular mean and *I*_*0*_*(κ)* is the modified Bessel function of order 0. The von Mises distribution approaches a Gaussian distribution in the limit where *κ* is large (in which case *κ* ~ 1/σ^2^, where *σ* is the standard deviation), but approaches a uniform distribution in the limit where *κ* goes to zero. The standard deviation is ill-defined for bimodal von Mises distributions^42^ and can lead to unintuitive results when used in data with low concentration (κ ~ 0) and poor directionality^43^. Thus, we did not calculate the standard deviation or the standard error as a measure of the uncertainty on the mean. Instead, we calculated a standard uncertainty *u* using the von Mises probability density function *pdf(θ)* so that the probability P(−*u* < *θ* < *u*) = 68%. In the limit where *κ* is large, the von Mises distribution resembles a Gaussian distribution and *u* approaches the standard deviation.

### Visualizing embryonic coronary microvasculature

**Figure 2:**
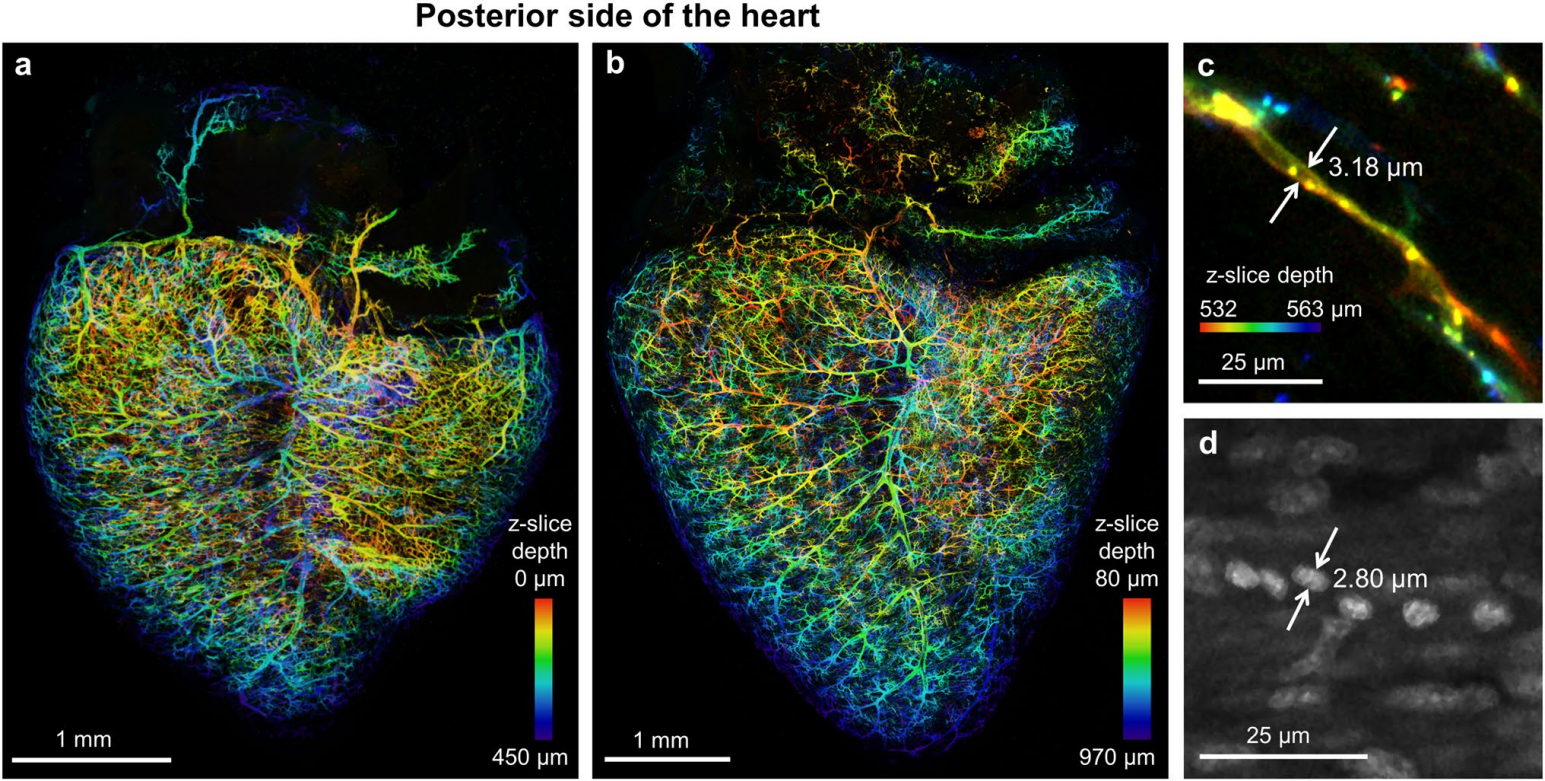
Highly developed, dense and organized quail coronary vasculature in the day 9 and 13 embryo. Vessels stained with DiI and color-coded based on depth in the 3D confocal image set. **(a)** Embryonic day 9. **(b)** Embryonic day 13. Posterior heart wall from the epicardium to the middle of the ventricular lumen. Two representative hearts shown with n = 4 hearts each for ED9 and ED13. Images colored with FIJI based on z-slice depth from confocal image stack. **(c)** Diameter of a vessel cross section after DiI perfusion. **(d)** Short diameter of blood cell nuclei as seen in DAPI images.

Day 9 and 13 embryonic hearts (n = 4 per developmental stage) showed a dense coronary network throughout the wall thickness previously unreported in the quail at these developmental stages (Fig. 2a,b). Our DiI perfusion and clearing technique resulted in high contrast images with continuous, highly connected vessels with no vessel leakage. Based on relative size of the smallest vessels (Fig. 2c) and the diameter of some blood cell nuclei found in the same sample (Fig. 2d), it is likely that the DiI perfusion enables imaging of vessels down to capillaries. LIMPID reduced light scattering which eliminated background signal from surrounding tissue and enabled intact heart imaging obviating sectioning and registration. Overall, the high image quality permitted automatic 3D vessel segmentation, which is essential to analyze volumetric images in experiments with a large number of animals per cohort.

### Embryonic coronary vasculature progressively changed orientation through the transmural depth of the ventricular wall

Vessel images were analyzed to determine vessel orientation through the left ventricular posterior free wall. The helical angle describes how vessels cross in and out of the horizontal plane, with circumferential vessels at 0°, and vessels that go from the apex to the base at 90°.

**Figure 3:**
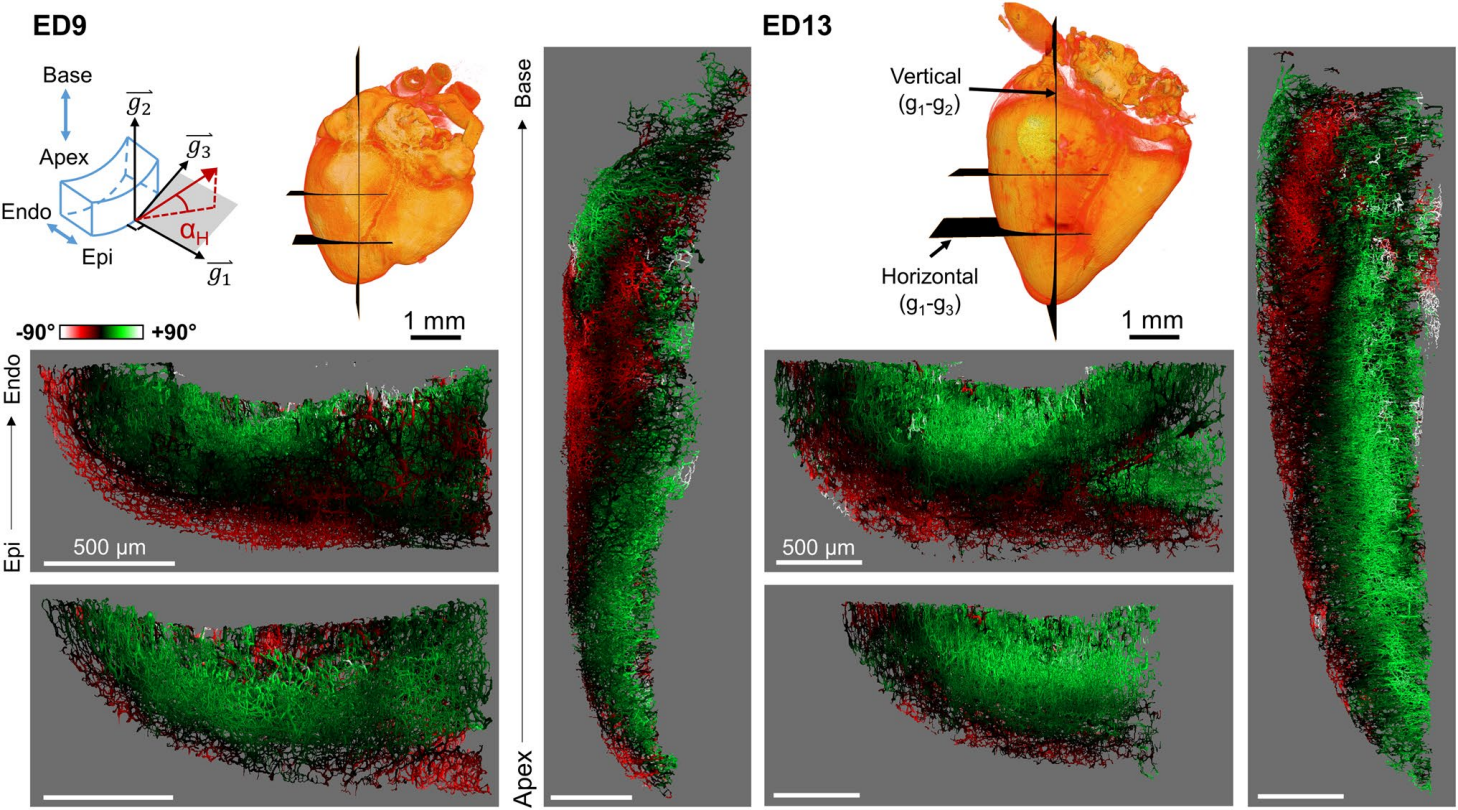
Mean coronary microvasculature orientation in the left ventricular posterior wall. Hearts at two developmental stages (left: ED9, right: ED13) with vessels color-coded based on the local mean helical angle α_H_, as defined by the coordinate vectors g_1_–g_2_–g_3_. In gray, cross-section views for each heart. Two horizontal cross-sections (g_1_–g_3_ plane) at different locations between the apex and the base. One vertical cross-section (g_1_–g_2_ plane) intersecting the ventricle from the apex to the middle of the left ventricular base. Volumetric heart surface images (orange) created from the DAPI images with the location of each cross-section indicated (black planes). Two representative hearts shown with n = 4 hearts each for ED9 and ED13. *Epi* Epicardium, *Endo* Endocardium. Images rendered in Amira with angles calculated in MATLAB.

To assess the orientation of the ventricular wall vessels at embryonic day (ED) 9 and ED13, the mean helical angle was calculated within a small moving window (approx. 136 × 136 × 175 μm), and each vessel was color-coded based on the result (Fig. 3, Supplemental videos 1, 2). As seen on the multiple cross-sections, the surface vessels started with a downward orientation toward the apex (helical angle is negative, color-coded red) before gradually approaching the horizontal plane deeper into the ventricular wall (0°, black), and continuing into the positive (green) toward the endocardium, with even some vessels becoming entirely vertical from apex to base (+ 90°, white) near the ventricular lumen (Fig. 3). This trend was detected in both ED9 and ED13 hearts, even though the overall heart shape matured by ED13 to become more like the adult quail heart.

**Figure 4:**
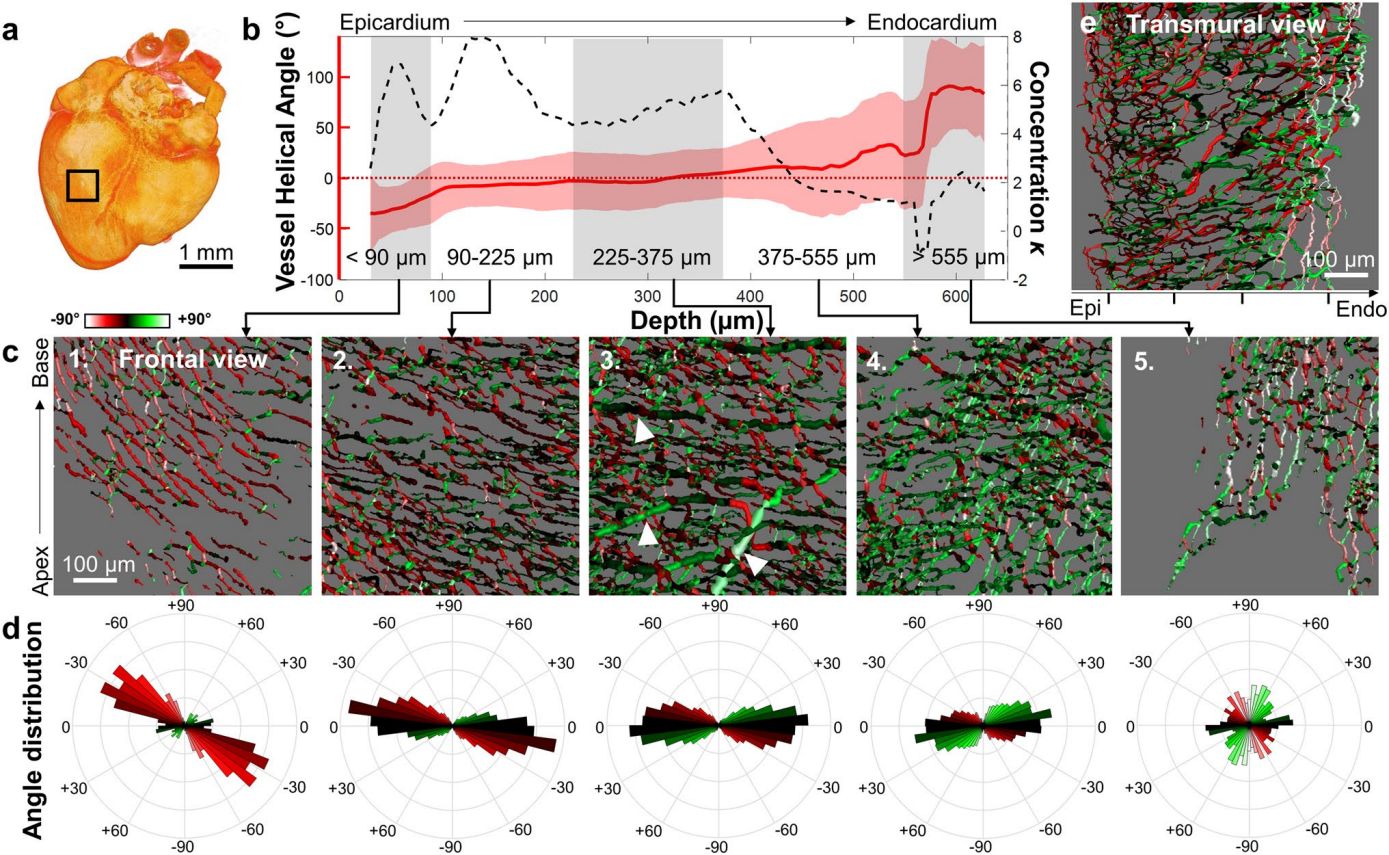
Quantifying vessel orientation as a function of depth at embryonic day 9. **(a)** Heart surface created from DAPI images with region-of-interest (ROI) indicated with black box. **(b)** Mean helical angle (red line, left y-axis) calculated at each depth with a moving window (+ /− 15 μm). Standard uncertainty shown as error bars. The concentration parameter *κ* was obtained from a von Mises fit performed at each depth (black dashed line, right y-axis). Layers of interest indicated with grey/white shaded regions. Helical angle of 0° indicated with a dashed red line. **(c)** Vessel images with color coding for helical angle at increasing depths (panels 1–5, depth range indicated in b). White arrows indicate large vessels. **(d)** Helical angle distribution matched to above panels in c (same color-scale). **(e)** Transmural cross-section of vessels from the epicardium (Epi) to the endocardium (Endo). Black tick marks separate the five regions in depth shown in b. One representative heart shown with n = 4 hearts for ED9. Images rendered in Amira with angles calculated in MATLAB.

**Figure 5:**
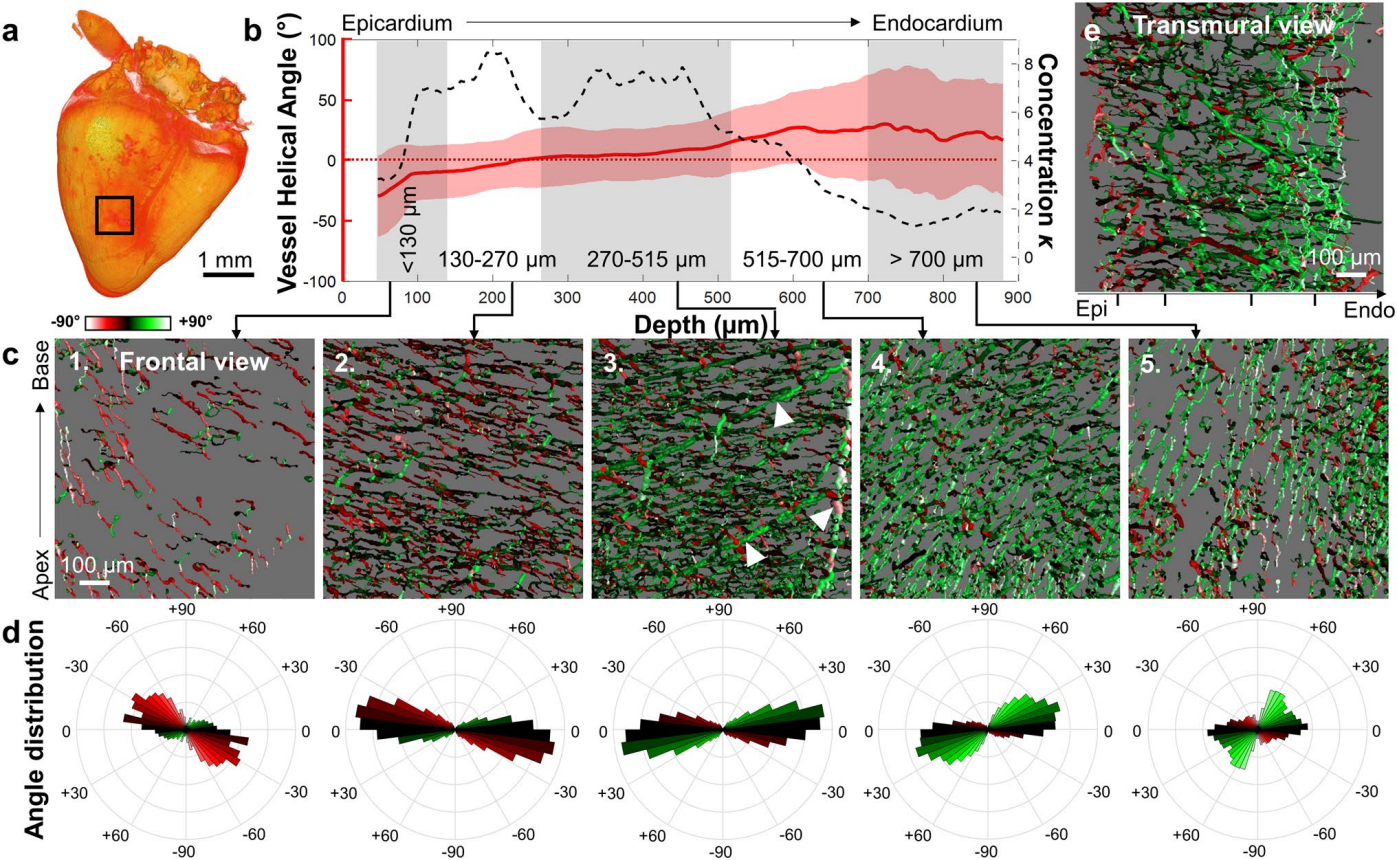
Quantifying vessel orientation as a function of depth at embryonic day 13. **(a)** Heart surface created from DAPI images with region-of-interest (ROI) indicated with black box. **(b)** Mean helical angle (red line, left y-axis) calculated at each depth with a moving window (+ /− 15 μm), with the standard uncertainty as error bars. The concentration parameter *κ* was obtained from a von Mises fit performed at each depth (black dashed line, right y-axis). Layers of interest indicated with grey/white shaded regions. Helical angle of 0° indicated with a dashed red line. **(c)** Vessel images with color coding for helical angle at increasing depths (panels 1–5, depth range indicated in **b**). White arrows indicate large vessels. **(d)** Helical angle distribution matched to above panels in (**c**) (same color scale used). **(e)** Transmural cross-section of vessels from the epicardium (Epi) to the endocardium (Endo). Black tick marks separate the five regions in depth shown in (**b**). One representative heart shown with n = 4 hearts for ED13. Images rendered in Amira with angles calculated in MATLAB.

To understand how individual microvessels contributed to the mean vessel orientation, we focused on a central region throughout the depth of the posterior left ventricular free wall at ED9 (Fig. 4) and ED13 (Fig. 5).

For the ROI indicated in Figs. 4a and 5a, the mean helical angle $\overline{{\alpha_{H} }}$ was calculated at all depths (Figs. 4b and 5b, left y-axis, red line). To quantify vessel angle distribution for each depth, the concentration parameter *κ* was obtained from the von Mises fit. High *κ* indicates vessel angles concentrated around the mean (i.e., vessels are aligned with each other), while low *κ* indicates vessels oriented in all directions equally (Figs. 4b and 5b, right y-axis, black dashed line). Five layers of the ventricular wall (Figs. 4b and 5b, gray/white shaded area) were identified for analysis based on the concentration parameter *κ*.

The most superficial layer, which starts at the epicardium and ends 90 μm into the myocardium for ED9 and 130 μm for ED13, was characterized by highly aligned vessels (high *κ*) with negative but rapidly changing helical angles (Figs. 4c and 5c, first panel). A second highly aligned layer (90–225 μm in depth for ED9, 130–270 μm for ED13) contained vessels oriented closer to the horizontal plane (color-coded black, Figs. 4c and 5c, second panel). Polar histograms of vessel angle distributions (Figs. 4d, 5d) showed that vessels in the first and second layers are oriented on average at − 27° and − 13° for ED9 (− 23° and − 12° for ED13). A decrease in vessel alignment (concentration *κ* diminished) marked the beginning of a third layer (ED9: 225–375 μm, ED13: 270–515 μm) where larger blood vessels were observed (Figs. 4c and 5c, white arrows). These larger vessels did not strongly align with the more numerous smaller vessels surrounding them. Finally, the fourth and fifth layers were defined as being between 375–555 μm and > 555 μm in depth at ED9 (515–700 μm and > 700 μm at ED13). Fourth layer vessels started to lose their alignment with each other (sudden decrease in *κ*) and the mean helical angle became more strongly positive (color-coded green, Figs. 4c and 5c, fourth panel). This organization continued into the fifth layer where vessels became more vertical with low alignment.

Most vessels are not strictly parallel to the heart surface but are often penetrating the ventricular wall, which is not reflected in the helical angle. As seen from the transmural view (Figs. 4e, 5e), individual vessels were more likely to be parallel to the heart surface near the epicardium and endocardium, with vessels in the middle traveling into the myocardium regardless of their helical angle.

In summary, coronary vessels in the left ventricular posterior wall were highly aligned with each other, especially in more superficial layers. The vessels gradually changed their orientation counterclockwise with depth at both ED9 and ED13. This alignment was detected in the microvasculature and was not strongly present in the larger coronary vessels.

### Cardiomyocytes in the embryonic heart progressively changed orientation through the transmural depth of the ventricular wall

Coronary vessels develop in close relationship with cardiomyocytes. An embryonic heart immunostained for MF20 confirmed that most DAPI-stained cell nuclei in our images were cardiomyocytes (Supplementary Fig. S1a). Another embryonic heart immunostained for NCAM to delineate the plasma membrane confirmed that embryonic cardiomyocytes contained one oblong nucleus, and the nuclear longitudinal axis corresponded to the cardiomyocyte longitudinal axis (Supplementary Fig. S1b). Thus, it was assumed that nuclei orientation in the ventricular wall represented the cardiomyocytes orientation for our analysis.

**Figure 6:**
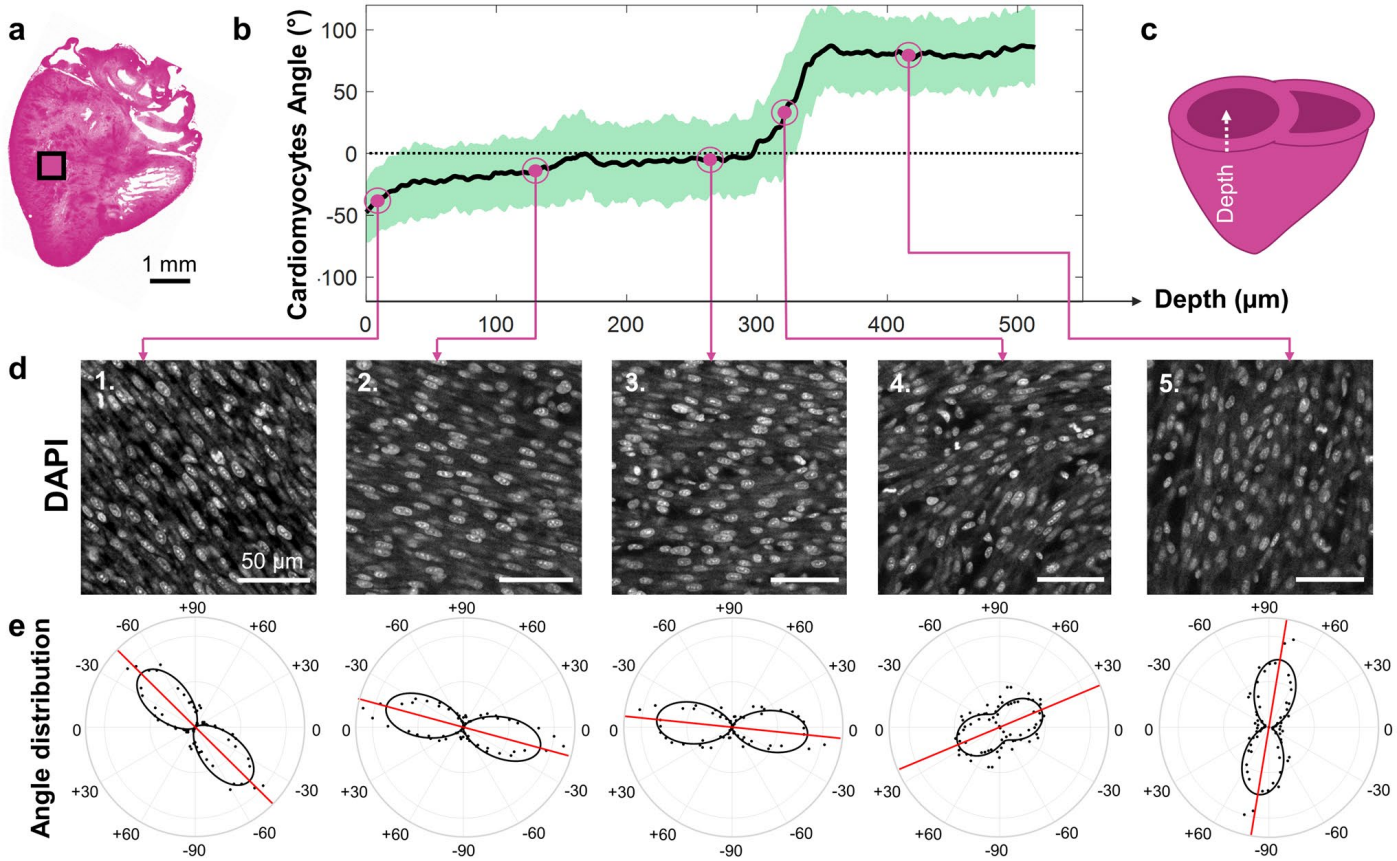
Quantifying cardiac nuclei orientation as a function of depth at embryonic day 9. **(a)** Frontal section of the heart created from DAPI images with region-of-interest (ROI) indicated with the black box. Image colorized in FIJI. **(b)** Mean cardiac nuclei orientation *μ* calculated at each depth *z* (black line) + /− standard uncertainty *u* with five representative points selected at different depths. Black dashed line indicates 0° (horizontal). **(c)** Heart ventricle diagram showing the depth axis direction into the left ventricular wall. **(d)** Five DAPI images corresponding to points in b showing individual cardiac nuclei at different orientations (images used for analysis are 360 × 360 μm). **(e)** Polar plot showing the corresponding angular distribution of the 2D Fourier transform spectrum (black dots), the von Mises fit (black line), and the mean cardiomyocyte angle (red line) for each depth shown in (**d**). One representative heart shown with n = 4 hearts for ED9.

Cardiac nuclei images were acquired for all hearts (ED9 and ED13, n = 4 hearts/group) immediately following DiI vascular imaging using a 20X objective. Images were acquired in the same ROI in the left ventricular posterior wall as for vessels. A representative heart is shown in Fig. 6, with the location of the ROI indicated in Fig. 6a.

The mean cardiomyocytes orientation $\overline{{A(\theta_{i} )}}$ was obtained using the 2D Fourier transform method. A von Mises fit was also performed to obtain the concentration parameter *κ,* from which the standard uncertainty *u* was calculated. The mean cardiomyocyte orientation through the ventricular wall depth is shown in Fig. 6b and depth axis orientation indicated in Fig. 6c. To demonstrate how cardiomyocytes orientation at each depth is obtained from individual confocal z-stack images, five images at different depths within the myocardium are presented in Fig. 6d. Nuclei were visibly elongated and aligned with each other, and their orientation gradually changed counterclockwise as depth increased, with an average orientation of − 40° near the epicardium and + 79° near the endocardium. For each image the angle distribution *A(θ*_*i*_*)* was plotted on a polar histogram (Fig. 6e, black dots), with the corresponding von Mises fit (Fig. 6e, black line) and mean cardiomyocyte orientation (Fig. 6e, red line). The von Mises fit shape indicated how aligned the cardiomyocytes were with each other, with highly aligned cells leading to an elongated “figure-eight” shape (Fig. 6e, black line), and low alignment leading to a more circular shape (Fig. 6e, fourth panel).

In summary, cardiomyocytes in the left ventricular posterior free wall already appeared elongated and aligned with each other at these early developmental stages, and their orientation gradually changed counterclockwise with depth at both ED9 and ED13.

### Cardiomyocytes were aligned with the coronary microvasculature at all depths throughout the left ventricle

To assess how coronary microvasculature is oriented with respect to the surrounding cardiomyocytes, we compared DiI-stained vascular images (10× magnification) with DAPI-stained nuclei images (20× magnification) for the same ROI in the left ventricular posterior wall. To compare the helical vessel angle (3D analysis) to cardiomyocyte orientation (2D analysis), the helical angle was projected onto the two-dimensional heart surface and became the projection angle *α*_*P*_.

**Figure 7:**
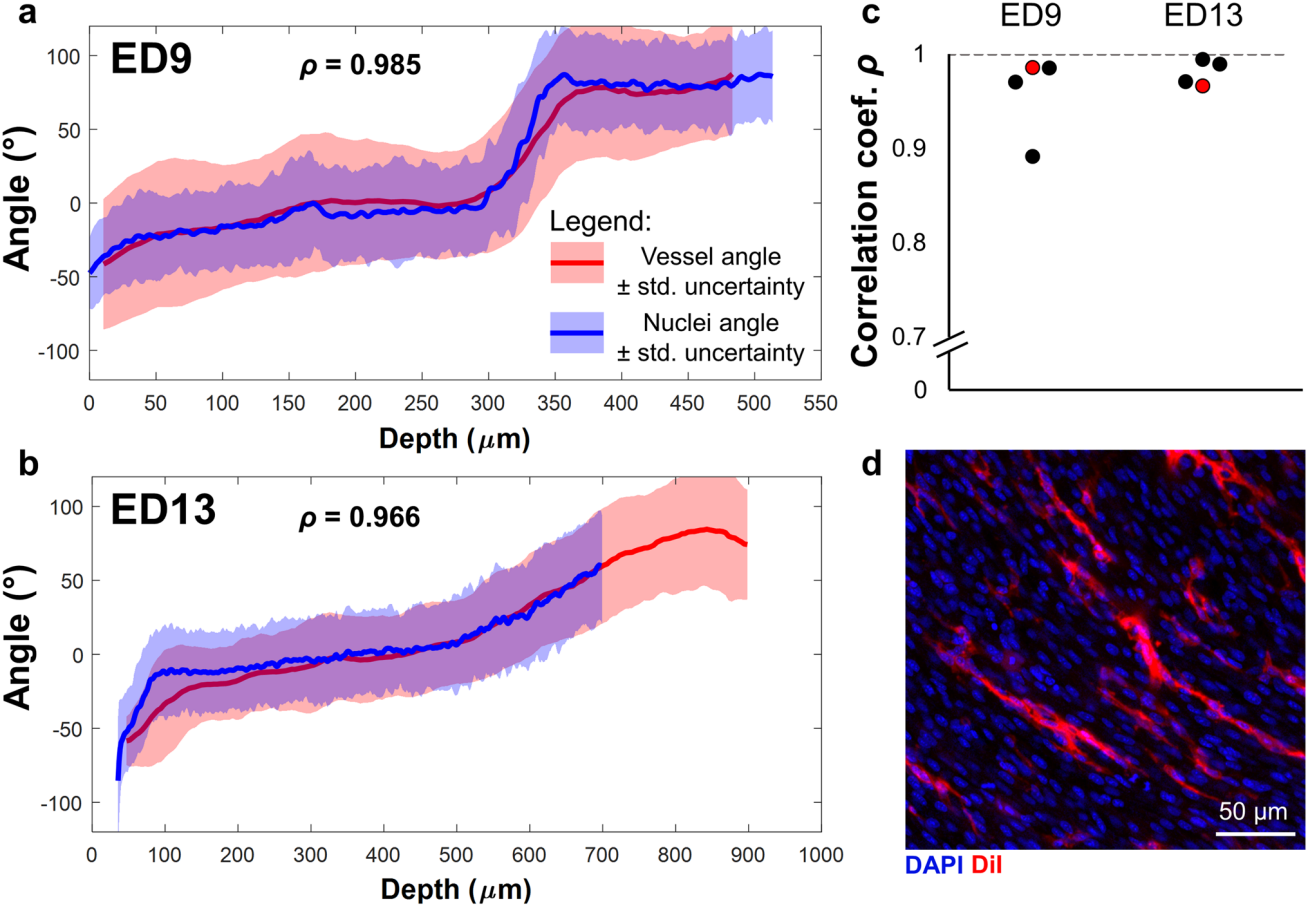
Cardiac nuclei and coronary vessels have the same orientation throughout the left ventricular wall. Mean projection angle for the vessels (red line) and mean orientation of cardiomyocyte nuclei (blue line) as a function of depth into the posterior left ventricular wall for **(a)** ED9 and **(b)** ED13, with standard uncertainty *u* as error bars. *ρ* is the Pearson’s correlation coefficient. **(c)** Pearson’s correlation coefficient between the mean vessel angle and the mean cardiomyocyte angle as a function of depth for all n = 4 ED9 hearts and all n = 4 ED13 hearts. Red data points indicate the hearts represented in (**a**) and (**b**). *ρ* = 1 is a perfect linear correlation. **(d)** Confocal microscopy image acquired at 20 × magnification in an ED13 heart with DAPI (blue) and DiI (red) signal. The DAPI signal was acquired at depth z = 496 μm, and the DiI signal was averaged over the range z = 482 μm to z = 511 μm. Image processed in FIJI.

The mean vessel projection angle $\overline{{\alpha_{P} }}$ (red) was plotted alongside the mean cardiomyocyte angle (blue) for all corresponding depths for a representative ED9 (Fig. 7a) and ED13 (Fig. 7b) heart. The two lines overlapped at all depths, demonstrating that cardiomyocytes were aligned with coronary microvasculature.

The Pearson’s correlation coefficient *ρ* was calculated to quantify how well the mean vessel orientation was correlated to the mean cardiomyocyte orientation throughout the ventricular wall, and thus how well these structures were aligned with each other.

Coefficients of *ρ* = 0.985 and *ρ* = 0.966 were obtained for the representative ED9 and ED13 hearts (Fig. 7a,b), indicating an almost perfect correlation between vessel and cardiomyocyte orientation. The coefficients of all other ED9 and ED13 hearts were also high (Fig. 7c) indicating that this relationship holds across the two different developmental stages. These results indicated that coronary vessels and cardiomyocytes are aligned even at early developmental stages. A representative image showing aligned coronary vessels and cell nuclei (Fig. 7d) also confirms these results.

### Coronary vessels orientation is consistent across individual hearts at both developmental stages

**Figure 8:**
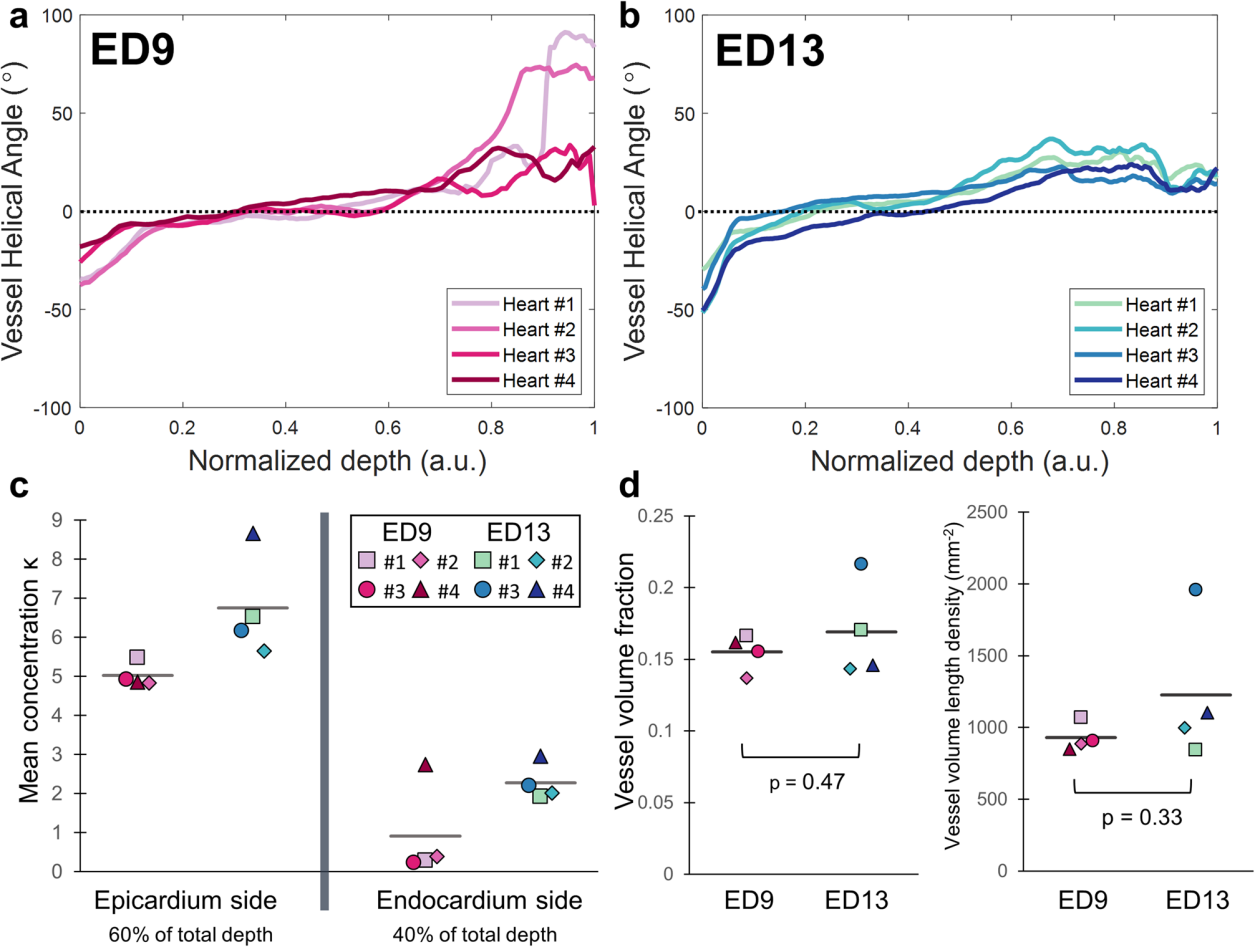
Comparison of mean vessel orientation, vessel alignment and vessel density across hearts. Mean coronary vessel helical angle as a function of depth in the posterior left ventricular wall for n = 4 hearts at **(a)** embryonic day (ED) 9, and **(b)** ED13. Depth across the ventricle wall is normalized for all hearts from 0 (closest vessels to epicardium) to 1 (closest vessels to endocardium). A.u. is arbitrary units. Black dashed line indicates the horizontal at 0°. **(c)** Concentration parameter *κ* averaged over the first 60% of the tissue starting from the epicardium (left) and the last 40% of the tissue until the endocardium (right) for all hearts at ED9 and ED13. Mean for each group indicated by gray line. **(d)** Vessel volume fraction (left) and vessel length density (right) for all hearts at ED9 and ED13. Mean for each group indicated by gray line. Groups compared using a two-tailed T-test assuming unequal variance.

The mean vessel helical angle as a function of depth was compared for all hearts at ED9 (Fig. 8a) and ED13 (Fig. 8b) to determine if coronary vessel organization was consistent across animals. The thickness of the posterior left ventricular wall was normalized from 0 to 1 to facilitate comparison (total wall thickness varied from 500 to 600 μm at ED9, and 800–1,000 μm at ED13).

At ED9, the coronary vessel orientation (mean helical angle) was similar across hearts near the epicardium and for most of the myocardium, but discrepancies across hearts were detected in the inner myocardium. In comparison at ED13, the mean helical angle was similar at all depths across all hearts.

To compare the degree of coronary vessel alignment across individual ED9 and ED13 hearts, the mean concentration parameter *κ* was calculated (Fig. 8c). As seen previously (Figs. 4b and Fig. 5b), the vessels were more aligned (concentration *κ* was higher) in more superficial layers of the myocardium, and less aligned (*κ* decreased) in the two inner most layers adjacent to the endocardium. Vessels were more aligned (higher *κ*) in ED13 hearts as compared to ED9, suggesting that coronary vessel alignment increased as the heart matured and became more organized. Additionally, we observe that values of κ are approaching zero near the endocardium at ED9 (Fig. 8c) which indicates random vessel orientation and an overall loss of directionality. As a result, the mean helical angle near the endocardium at ED9 (Fig. 8a) behaves erratically and is not consistent from heart to heart. However, these are qualitative observations and a statistical test was not performed to compare the mean concentration parameter *κ* between ED9 and ED13 hearts, since *κ* could not be assumed to be normally distributed, and *κ* was a measure of variance. We could not find a statistical test appropriate for this situation.

In summary, the coronary microvasculature showed strong alignment, with the left ventricular vessels rotating counterclockwise as a function depth. Vessels closer to the epicardium (60% of wall thickness) may have been more strongly aligned. Vessels may have become more aligned with increasing developmental stage (ED13 as compared to ED9).

### Vessel density remained the same between two developmental stages

To further quantify vessel organization in the developing ventricular wall, vessel density was calculated at both ED9 and ED13 using two methods: vessel volume fraction (no units) and vessel length density (units of mm^−2^) (Fig. 8d). The accuracy of the vessel volume fraction can be negatively affected by imaging conditions such as overexposed vessels, while the accuracy of the vessel length density is dependent on proper segmentation and skeletonization of the vessels. Thus, both methods were employed to counterbalance each method’s limitations. Both methods indicated no difference in vessel density as a function of developmental age. A two-tailed T-test assuming unequal variance between groups showed no statistically significant differences (*p* = 0.47 and *p* = 0.33 for volume fraction and vessel length density respectively). Thus, coronary vessels did increase in number in the growing ventricular wall, but the overall vessel density remained the same at ED9 and ED13.

## Discussion

In the current study, we quantified coronary organization shortly after the start of coronary circulation, an intermediate developmental stage which is rarely studied. Early coronary vascular formation has been the focus of many investigations^8–13^. The development of the large coronaries and congenital anomalies has also been a major research focus because of its clinical significance^44–46^. In contrast the intermediary stages when the ventricular walls rapidly thicken and the microvasculature develops within the myocardium has been addressed in few studies^23,47,48^, leaving many fundamental questions unanswered. These stages were the focus of this study. This developmental period is challenging to access in intact hearts due to the thickness and density of the myocardium. We deployed a combination of techniques and algorithms such as DiI perfusion, optical clearing, automated segmentation and directional statistics to visualize the coronary vessels down to the capillaries and quantify their orientation with respect to the surrounding cardiomyocytes. Using our toolset, we provided quantitative data to serve as a baseline for future studies of disease models.

The spatial organization of the developing coronaries had not been quantified, with previous studies conducted in turkeys, chickens, mice and humans focusing on capillary density^8,10–13,44,45^. Our recent study in quails^23^ uncovered highly aligned coronary microvessels at the newly septate embryonic heart surface instead of a plexus of coronaries as in preceding stages^49,50^. This strong alignment disappeared when the embryos were dosed with ethanol in a model of prenatal alcohol exposure, but the vessel alignment in neither the healthy nor the diseased hearts were quantified. In the present study, we used a novel staining and optical clearing protocol (LIMPID^41^) to visualize both the coronary vessels and cardiomyocytes in the same hearts. We also expanded on the previous qualitative observations by quantifying the organization of the coronary vessels in the free wall of the left ventricle for the first time. We discovered a well-developed and highly organized microvascular network as early as ED9, a stage soon after the connection of the coronary vessels to the aorta^48^. The vessels progressively changed their orientation counterclockwise from the epicardium to the endocardium. The variance in vessel alignment (parameter κ) was also quantified. Such a detailed quantitative analysis had not been performed for the coronary microvasculature in embryonic or adult animal models, or in human hearts before.

The vessel orientation followed similar trends at ED9 and ED13 (Fig. 8a,b). While the progressive change in vessel orientation was similar across all hearts at both stages, a higher variability across hearts and loss of directionality was seen in the helical angle toward the lumen at ED9 (Fig. 8c). This variability can be explained by the ongoing compaction and maturation of the trabeculae occurring at this stage^39^. However, this loss of directionality was not observed in the mean projection angle, which continued to follow the orientation of the cardiomyocytes (Fig. 7a,c). In the older embryonic stage (ED13), compaction is largely complete and therefore the more stable myocardial environment^40^ leads to more consistent orientation of the microvessels across individual hearts. As a follow up to this observation, future studies could explore how cellular level maturation markers correlate with vessel organization^51^.

Even though an increasing demand is put on the heart as the vasculature expands and the embryo grows, we found no change in vessel density in the left ventricular free wall between ED9 and ED13. This finding suggests that myocardial oxygen demand was stable during this time period and that wall thickness increases account for the increased cardiac output needed for older embryos. In the future, a detailed study of local density variations, cardiomyocyte maturation and hypoxia throughout the entire heart may reveal subtler changes in specific heart regions.

The coronary vasculature was found to be strongly aligned with the surrounding cardiomyocytes throughout the ventricular wall consistent with qualitative observations from previous studies. An SEM study of corrosion casts of the rabbit ventricle indicated that capillaries “run generally in parallel with cardiac muscle fibers^52^,” with similar results obtained in humans^53^. Another study detected both ventricular cardiomyocytes and coronaries running parallel to each other using DAPI and fluorescently labeled lectin (wheat germ agglutinin; WGA) that bind to plasma membranes in thick adult heart sections of various animals^28^. While many capillary parameters were assayed (e.g., capillary length density, total capillary length, diffusion radius, capillary surface area density, and total myocardial capillary surface area), the orientation of the capillaries with respect to the cardiomyocytes was neither assessed over large volumes nor quantified. In our study, microvascular orientation was quantified and had a strong correlation with cardiomyocyte orientation throughout the ventricular wall, which indicates strong alignment already at these developmental stages. The coronaries and cardiomyocytes had the same gradual change in orientation as the cardiac muscle fibers in the adult heart, which are known to rotate within the ventricle wall in a counterclockwise helical pattern^33^.

Alignment between the coronary microvasculature and the cardiomyocytes may be optimal for heart function during development. In a study focusing on the microvascular arrangement around skeletal muscle fibers, investigators found that arterioles and venules ran parallel with the length of the skeletal muscle fibers^27^. To study revascularization after skeletal muscle injury, they ligated the right femoral artery. The vasculature rapidly regenerated but failed to replicate the normal morphology or hemodynamic function and resulted in abnormal oxygen saturations in the capillaries. Therefore, the organized linear arrangement of the microvasculature is critical for appropriate skeletal muscle perfusion and may be similarly important for cardiac muscle.

The strong alignment between cardiomyocytes and coronary vessels in the healthy developing heart suggests a correlation between cardiomyocyte disarray and disorganized coronary vasculature in disease. Cardiomyocyte disarray has been observed in the mouse embryo as a precursor to hypertrophic cardiomyopathy^32^ and could contribute to a disorganized vasculature by affecting the maturation of the vascular plexus. In a previous study, we observed abnormal coronary microvascular alignment in the ventricular wall in a model of fetal alcohol spectrum disorders^23^. These abnormalities included chaotic microvasculature patterns and regions of missing vasculature. Highly aligned microvessels may provide optimal oxygen and nutrient transfer to the heart and aligned cardiomyocytes may be necessary for normal contractile patterns. Disarray in one of those two systems could reflect disarray in the other or impede development and proper function of either. The results presented in the current study indicate strong links between cardiomyocytes arrangements and coronary development, and further studies should consider the involvement of both systems in diseases.

Our study comes with certain limitations which should guide the interpretation of our results. The DiI injection technique allowed us to fill the entire coronary circuit including arterioles, capillaries and venules using gentle pressure. However, this amount of pressure may have opened shunts and beds that could be closed under different physiological conditions. Additionally, we predict that our vessel images included arterioles and venules, but we did not distinguish them in this study. In future studies, we plan to differentiate arterioles from venules using specific markers including antibodies for Tie2 expressed in veins^54,55^, and antibodies for smooth muscle cells found densely encircling the arteriole walls^56^. Finally, because the cardiomyocyte orientation was analyzed in 2D, a projection was used to compare to the coronary vessels. The microscope objective used for cardiomyocyte imaging (20×) also did not have sufficient working distance to image through the whole thickness of the ventricular wall at ED13 (Fig. 7b). In order to image the cardiomyocytes at all depths with high axial resolution and to characterize their orientation in 3D, new imaging and statistical analysis strategies will be part of our future work.

Moving forward, the imaging and analysis toolset presented in this study will be used to characterize coronary vessel and cardiomyocyte distribution in diseased embryonic hearts. The role of the microvasculature in the formation of CHDs is understudied, and little is known about the effects of disorganized microvasculature on heart function or possible impacts on adult diseases. In addition to studying development, our method can be expanded to a wide array of other applications such as evaluating the organization of vessels in engineered cardiac tissue or improving our understanding of coronary microvascular disease. Our DiI perfusion, optical clearing protocol and data analysis toolset can also be adapted for mature hearts of various species and can be used to study other vascular beds throughout the body including the skeletal muscle, brain, liver, gut and kidney.

## Materials and methods

### Fluorescent dye preparation (DiI)

The fluorescent lipophilic carbocyanine dye DiI was used to stain coronary vessel walls in the embryonic quail heart. The DiI solution was prepared as previously published^57^. In summary, a stock solution was made using 100 mg of DiI crystals (D282, Invitrogen, Thermo Fisher Scientific, Waltham, MA, USA) diluted in 16.7 ml of 100% ethanol, which was then rocked overnight while protected from light. Immediately before each experiment 100 μl of the DiI stock solution was added to 25 ml of diluent composed of phosphate-buffered saline (PBS) mixed at a 1:4 ratio with a 5% glucose solution, then sonicated for 1 min.

### Avian model

Fertilized quail eggs (*Coturnix coturnix japonica*, Northwest Heritage Quail, Pullman, WA, USA) were incubated at 38 °C for 9 or 13 days, corresponding approximately to Hamburger and Hamilton (HH) stages 36 and 42^58,59^ (n = 4 embryos per developmental stage). ED9 is soon after the start of coronary circulation and during ventricular compaction, while ED13 is at the end of ventricular wall compaction. Eggs were randomly assigned to the ED9 or ED13 groups when incubation first started. The embryos’ sex was not identified.

### Coronary vasculature perfusion

After removal of the embryo from the egg, a fine glass capillary tube was inserted into the aorta near the junction with the left and right brachiocephalic arteries (Fig. 1a), without puncturing the aortic valve. Approximately 0.5 ml of PBS was perfused through the heart followed by 0.5–1 ml of the DiI solution. Hearts were fixed overnight in 4% paraformaldehyde at 4 °C, then stained for 48 h with DAPI (10 mM solution in water diluted 1:500 in PBS without detergent). Optical clearing was performed for 2–4 days at room temperature using LIMPID^41^. Following this step, improperly perfused hearts were excluded from the study. All others were included.

### Imaging protocol

Images were acquired using an inverted confocal microscope (SP8 with HyVolution 2, Leica Microsystems Inc., Buffalo Grove, IL, USA). Hearts were positioned posterior face down between two glass cover slips while immersed in mineral oil. For vessel imaging, a 10×/0.40 NA air objective was used (excitation: 561 nm, emission: 572–614 nm and 660–728 nm). Two emission channels at different wavelength ranges were used and recombined during post-processing to avoid pixel saturation. DAPI signal was simultaneously acquired (excitation: 405 nm, emission: 416–490 nm). Pixel size was approximately 2.27 × 2.27 × ~ 4 μm (x, y, z). The exact pixel size in z depended on the sample. Optical sectioning was 7.2 μm. Excitation compensation was used to progressively increase the excitation laser power at higher depths within the sample. Excitation intensity and excitation compensation with depth were manually adjusted at each depth for each sample in order to obtain an optimal signal-to-noise ratio without overexposing nuclei or vessels.

To image the cardiac nuclei, a 20×/0.75 NA objective set for glycerol immersion was used, with the same excitation and emission wavelengths as for 10× imaging. Images were collected over a 700 × 700 μm region-of-interest (ROI) on the posterior wall of the left ventricle, with pixel size of 177.6 × 177.6 nm × ~ 1.5 μm (x, y, z). Images were acquired to cover the thickness of the posterior ventricle wall, or as deep as the objective working distance would allow (500–600 μm). Optical sectioning was 3.12 μm. Excitation compensation was also used.

### Image analysis

#### Coronary vessel image analysis

To segment and skeletonize vessels, the microscope images were automatically stitched and imported into MATLAB (MathWorks, Natick, MA, USA) where the two DiI emission channels were combined into one high dynamic range image, then imported into Amira 6.5 (Thermo Fisher Scientific, Waltham, MA, USA), where the background was subtracted and a median filter was applied. An intensity-based threshold was used to create a mask identifying all vessels (Fig. 1d). Manual corrections were made to the mask to fill the lumen of the large coronary arteries if they appeared as hollow (Supplementary Fig. S2). The auto-skeleton function created a series of points, nodes and segments describing every vessel’s position (Fig. 1e). Further analysis was conducted in MATLAB.

A custom-written MATLAB code was used to determine the distance between each vessel and the heart’s surface. DAPI images acquired at 10× were used to determine the surface’s position. The Euclidean distance between all vessel-points and all surface-points was calculated, and the smallest distance was identified as the depth of each point. Similarly to a method outlined in Garcia-Canadilla et al.^32^ a reference axis system *g*_*1*_, *g*_*2*_, *g*_*3*_ was defined for each vessel-point. The vector connecting a vessel-point *(x*_*p*_*, y*_*p*_*, z*_*p*_*)* to its nearest surface-point *(x*_*s*_*, y*_*s*_*, z*_*s*_*)* was identified as *g*_*1*_, (Fig. 1f).

2 $$\overrightarrow {{g_{1} }} = \frac{{(x_{s} - x_{p} ,y_{s} - y_{p} ,z_{s} - z_{p} )}}{{\sqrt {(x_{s} - x_{p} )^{2} + (y_{s} - y_{p} )^{2} + (z_{s} - z_{p} )^{2} } }}$$

A unit vector *V*_*AB*_ linking the apex to the middle of the left ventricle base was manually identified as a vertical reference axis. This axis projected onto the heart’s surface was defined as *g*_*2*_ for each vessel-point (Fig. 1f), and *g*_*3*_ was defined to be perpendicular to both *g*_*1*_ and *g*_*2*_.

3 $$\overrightarrow {{g_{2} }} = \overrightarrow {{g_{1} }} \times \left( {\overrightarrow {V}_{AB} \times \overrightarrow {g}_{1} } \right)$$

4 $$\overrightarrow {{g_{3} }} = \overrightarrow {{g_{2} }} \times \overrightarrow {{g_{1} }}$$

A vector *V*_*v*_ linking the start and end node of each vessel-segment was used to calculate the vessel’s orientation (Fig. 1e,f, red arrow). The helical angle *α*_*H*_ in radians was calculated as follows^32^:

5 $$\alpha_{H} = {\raise0.7ex\hbox{$\pi $} \!\mathord{\left/ {\vphantom {\pi 2}}\right.\kern-\nulldelimiterspace} \!\lower0.7ex\hbox{$2$}} - \cos^{ - 1} \left[ {\frac{{\overrightarrow {V}_{v} \cdot \overrightarrow {{g_{2} }} }}{{\left\| {\overrightarrow {V}_{v} } \right\| \cdot \left\| {\overrightarrow {{g_{2} }} } \right\|}}} \right]$$

The projection angle *α*_*P*_ between *g*_*3*_ and the vessel-vector *V*_*p*_ (obtained by projecting *V*_*v*_ onto the heart’s surface) was also calculated to compare the orientation of vessels and cardiomyocytes:

6 $$\overrightarrow {V}_{p} = \overrightarrow {{g_{1} }} \times \left( {\overrightarrow {V}_{v} \times \overrightarrow {{g_{1} }} } \right)$$

7 $$\alpha_{P} = \cos^{ - 1} \left[ {\frac{{\overrightarrow {V}_{p} \cdot \overrightarrow {{g_{3} }} }}{{\left\| {\overrightarrow {V}_{p} } \right\| \cdot \left\| {\overrightarrow {{g_{3} }} } \right\|}}} \right]$$

#### Cardiomyocytes image analysis

Cardiomyocytes images were processed in MATLAB following an algorithm outlined in Marquez^43^. A vector linking the apex to the middle of the left ventricle base was manually identified as a vertical reference axis. A 2D Fourier transform was performed onto each DAPI image, resulting in a spectrum *F(k*_*x*_*, k*_*y*_*)*, where *k*_*x*_ and *k*_*y*_ are horizontal and vertical spatial frequencies. To calculate the orientation of the elongated disk formed by *F(k*_*x*_*, k*_*y*_*),* it was multiplied by a series of masks *h*_*θi*_*(k*_*x*_*, k*_*y*_*)* (Fig. 1i) that selected sections of *F(k*_*x*_*, k*_*y*_*)* corresponding to different angles *θ*_*i*_ in increment of 5° from 0° to 180°.

8 $$A(\theta_{i} ) = \sum\limits_{{k_{y} }}^{{N_{y} }} {\sum\limits_{{k_{x} }}^{{N_{x} }} {\left| {F(k_{x} ,k_{y} )} \right|^{2} \cdot h_{{\theta_{i} }} (k_{x} ,k_{y} )} }$$

where *A(θ*_*i*_*)* is the angular amplitude of *F(k*_*x*_*, k*_*y*_), and *N*_*x*_ and *N*_*y*_ are the number of pixels in the x and y direction. This amplitude was then normalized so that its minimum value was zero and the area under the curve was 1.

#### Coronary vessel density

The vessel volume fraction was calculated by taking the number of segmented vessel pixels divided by the total number of pixels in the ROI. The vessel length density was taken as the sum of the lengths of all vessel-segments within the ROI, in units of mm, divided by the volume of the ROI, in units of mm^3^.

#### Blinding

Researchers could not be blinded to the heart’s developmental stage during analysis due to morphological differences between ED9 and ED13. All image analysis was automated to remove bias.

### Statistical analysis

#### Circular mean

The mean helical angle and mean cardiomyocyte angle were calculated by vector summation in the complex plane^42^. Since both vessels and nuclei have axial symmetry (orientations of 1° and 181° are the same), the traditional formula for the circular mean was modified as follows^42^:

9 $$\alpha_{i}^{double} = 2 \cdot \alpha_{i}$$

10 $$\overline{{\alpha_{double} }} = \frac{1}{N}\sum\limits_{i = 1}^{N} {\exp (j\alpha_{i}^{double} )}$$

11 $$\overline{\alpha } = \frac{1}{2} \cdot \overline{{\alpha_{double} }}$$

where *α*_*i*_ are individual angles in radians, $\overline{\alpha }$ is the circular mean, *N* is the total number of angles being averaged, *j* is the imaginary unit j^2^ = −1.

#### Von Mises distribution

A bimodal von Mises probability distribution function (pdf) is given in Eq. (1). To fit our data to a bimodal von Mises distribution, the following function is minimized to calculate the experimental concentration *κ*_*exp*_^43^:

12 $$\mathop {\min }\limits_{\kappa } \left( {\sum\limits_{i = 1}^{N} {\left( {\alpha_{i} - \left. {pdf(\theta_{i} )} \right|_{{\mu = \overline{\alpha } }} } \right)^{2} } } \right) \to \kappa_{\exp }$$

#### Pearson’s correlation coefficient

The Pearson’s correlation coefficient was calculated in MATLAB between the mean projection angle for the vessels $\overline{{\alpha_{P} }}$, and the mean nuclei orientation $\overline{{A(\theta_{i} )}}$ as a function of depth for the same ROI in each heart. Each variable was first resampled to 200 data points in depth using one-dimensional linear interpolation to prevent variations in coefficient due to different numbers of data points.

#### Error bars

The circular variance or standard deviation are ill-defined for bimodal von Mises distributions^42^. We calculated a standard uncertainty *u* using the von Mises probability density function *pdf(θ)* centered at μ = 0 so that the probability P(−*u* < *θ* < *u*) = 68%. In the limit where *κ* is large, *u* approaches the standard deviation. However, this measure is also valid when *κ* is not large, which cannot be assumed when calculating the standard deviation directly.

#### Statistical tests

Statistical testing was performed in Excel. To compare the vessel density between embryos at ED9 and ED13 (n = 4 hearts/group), a one-tailed F-test was performed to determine if the variance of the two groups were equal. This test provided a *p* value > 0.05 and the null hypothesis was not rejected. A two-tailed T-test assuming unequal variance was then performed to compare the mean of the ED9 and ED13 groups. It was assumed that individual hearts are independent from each other and follow a normal distribution. Formal sample size and power calculations were not conducted before commencing this study since this was an exploratory study focused on imaging and analysis tool development.

### Ethics statement

Quail embryos typically hatch at ED17. Institutional Animal Care and Use Committee (IACUC) approval was not required for this study as the Policy for use of Avian embryos at Case Western Reserve University states “if embryos will be sacrificed prior to 3 days before hatching, the research will not be subject to IACUC review.” All experiments were performed in accordance with relevant guidelines and regulations.

## Supplementary information

Supplementary information

Supplementary video 1

Supplementary video 2

## Data Availability

The data that support the findings of this study are available from the corresponding author upon reasonable request.

## Code availability

The custom computer codes (MATLAB) used in this study are available from the corresponding author upon reasonable request.
