# Supplementary material for: Three-dimensional alignment of microvasculature and cardiomyocytes in the developing ventricle
Source: Sci Rep. 2020 Sep 11;10:14955. doi: 10.1038/s41598-020-71816-y (PMC7486945; doi:10.1038/s41598-020-71816-y)
Supplement: Supplementary file 1 — Supplementary information [file 41598_2020_71816_MOESM1_ESM.docx]

**Supplementary Materials**

**Full title:** Three-dimensional alignment of microvasculature and cardiomyocytes in the developing ventricle

**Authors:**

Maryse Lapierre-Landry^1†^, PhD, Hana Kolesová^2,3†^, PhD, Yehe Liu^1^, MS, Michiko Watanabe^2^, PhD, Michael W. Jenkins^1,2^*, PhD

^1^Department of Biomedical Engineering, School of Medicine, Case Western Reserve University, USA;

^2^Department of Pediatrics, School of Medicine, Case Western Reserve University, USA;

^3^Institute of Anatomy, First Faculty of Medicine, Charles University, Prague, Czech Republic

^†^Co-first authors
*Corresponding author

**Corresponding author:**Michael W. Jenkins.
michael.jenkins@case.edu
Wood Building WG28
2109 Adelbert Road,
Cleveland, OH 44106
USA

**Supplementary methods:**

One heart was immunostained for NCAM, a transmembrane protein found on the plasma membrane of cardiomyocytes^1–3^, to confirm that the longitudinal axes of the elongated oval cardiomyocyte nuclei align with the longitudinal axes of the cell. Mouse monoclonal NCAM1 (5e) antibody were applied in 1:200 dilution overnight, followed by secondary anti-mouse IgG AlexaFluor 594 (Invitrogen) at 1:200 dilution overnight. The monoclonal antibody 5e developed by Dr. Urs Rutishauser and laboratory at CWRU and was obtained from the Developmental Studies Hybridoma Bank, created by the NICHD of the NIH and maintained at The University of Iowa, Department of Biology, Iowa City, IA 52242. The heart was then DAPI stained and optically cleared as described above. Two hearts were immunostained with MF20 antibody to visualize the myosin heavy chain II in cardiomyocytes. MF20 mouse monoclonal antibody (AB_2572894, Invitrogen) was diluted 1:200 in PBS and incubated overnight. Then secondary antibody Goat anti-mouse Alexa 488 IgG (H+L) (A28175, Invitrogen) was applied (1:200). The heart was then DAPI stained and optically cleared as described above.

**References:**

1. Burroughs, C. L., Watanabe, M. & Morse, D. E. Distribution of the neural cell adhesion molecule (NCAM) during heart development. *Journal of molecular and cellular cardiology* **23**, 1411–1422 (1991).

2. Watanabe, M., Frelinger, A. L. & Rutishauser, U. Topography of N-CAM structural and functional determinants. I. Classification of monoclonal antibody epitopes. *J Cell Biol* **103**, 1721–1727 (1986).

3. Frelinger, A. L. & Rutishauser, U. Topography of N-CAM structural and functional determinants. II. Placement of monoclonal antibody epitopes. *J Cell Biol* **103**, 1729–1737 (1986).

**Supplementary figures:**


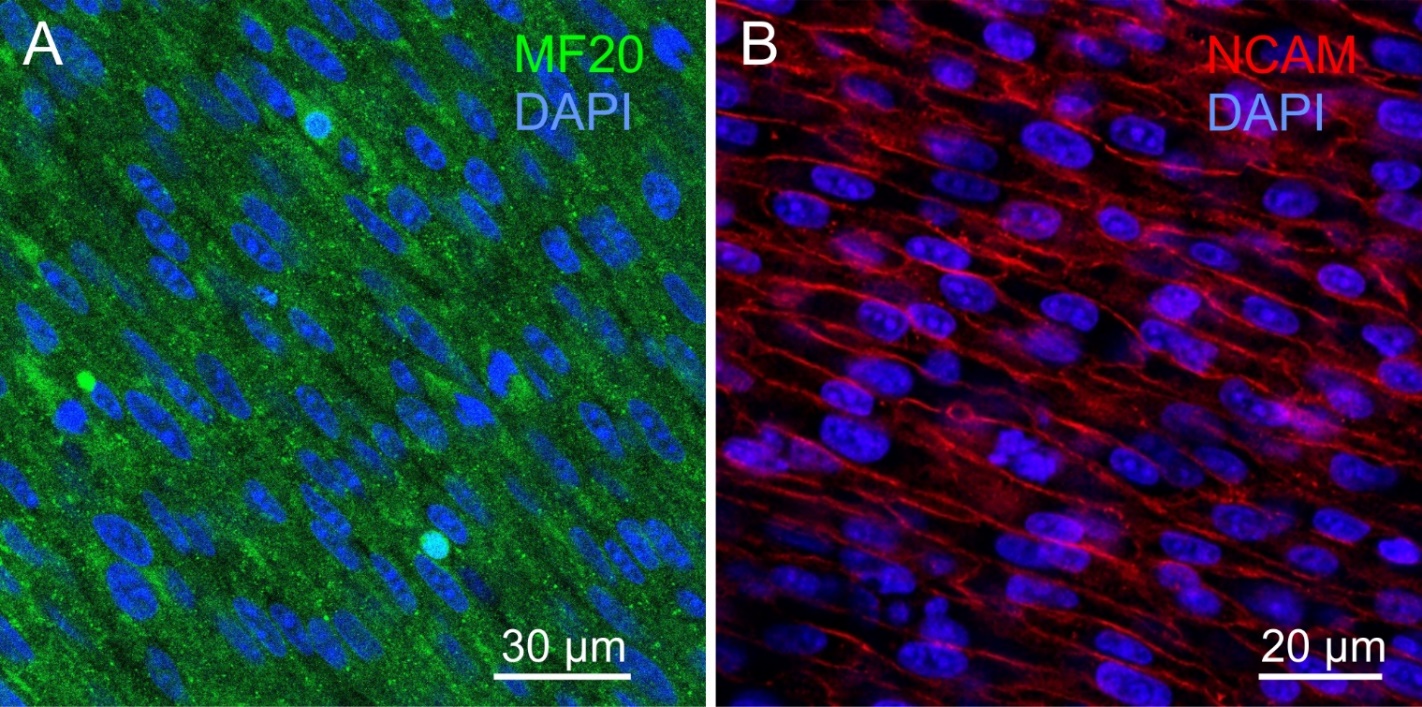


**Supplementary figure 1: Cardiac nuclei orientation align with the orientation of the cardiomyocytes.** **A)** An embryonic heart (ED9) immunostained with MF20 confirmed that most cell nuclei seen were cardiomyocytes. **B)** An embryonic heart (ED9) was immunostained with NCAM antibody to identify the plasma membrane, and DAPI to identify the nuclei. N=1 for each experiment.


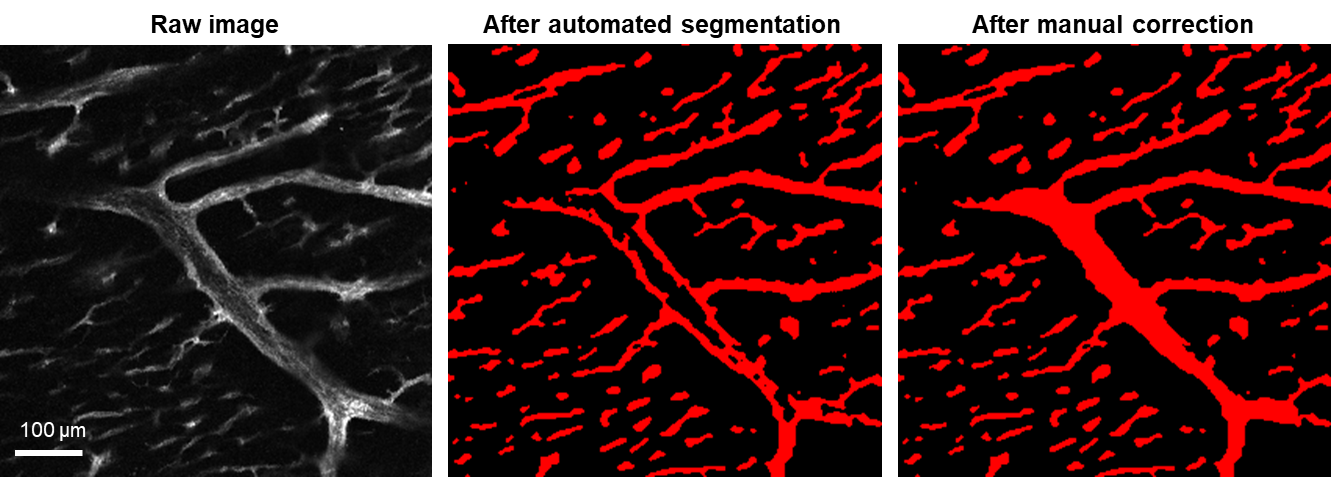


**Supplementary figure 2:** **Automated vessel segmentation with manual correction.** **Left:** Original DiI fluorescence image showing blood vessels. The lumen of large blood vessels is not stained as much as the vessel walls. **Middle:** Vessel mask following intensity-based thresholding. The lumen of a large vessel has been excluded from the vessel mask. **Right:** The vessel mask is manually corrected to include the lumen of large vessels. No manual correction is applied for smaller vessels.

**Video captions:**

**Video 1:** **Mean orientation of the coronary microvasculature in the posterior wall of the left ventricle at ED9.** Posterior wall of the left ventricle with vessels color-coded based on the local mean helical angle α_H_. Cross-section progressively moving from the epicardium to the endocardium.

**Video 2:** **Mean orientation of the coronary microvasculature in the posterior wall of the left ventricle at ED13.** Posterior wall of the left ventricle with vessels color-coded based on the local mean helical angle α_H_. Cross-section progressively moving from the epicardium to the endocardium.
